# Diffusivity Maximum in a Reentrant Nematic Phase

**DOI:** 10.3390/ijms13067854

**Published:** 2012-06-21

**Authors:** Tillmann Stieger, Marco G. Mazza, Martin Schoen

**Affiliations:** 1 Stranski-Lab for Physical and Theoretical Chemistry, Berlin Institute of Technology, 135 June 17th Street, Berlin 10623, Germany; E-Mails: tillmann.stieger@mailbox.tu-berlin.de (T.S.); martin.schoen@tu-berlin.de (M.S.); 2 Department of Chemical and Biomolecular Engineering, North Carolina State University, 911 Partners Way, Raleigh, NC 27695, USA

**Keywords:** reentrant phase, nematic, dynamics, diffusion

## Abstract

We report molecular dynamics simulations of confined liquid crystals using the Gay–Berne–Kihara model. Upon isobaric cooling, the standard sequence of isotropic–nematic–smectic A phase transitions is found. Upon further cooling a reentrant nematic phase occurs. We investigate the temperature dependence of the self-diffusion coefficient of the fluid in the nematic, smectic and reentrant nematic phases. We find a maximum in diffusivity upon isobaric cooling. Diffusion increases dramatically in the reentrant phase due to the high orientational molecular order. As the temperature is lowered, the diffusion coefficient follows an Arrhenius behavior. The activation energy of the reentrant phase is found in reasonable agreement with the reported experimental data. We discuss how repulsive interactions may be the underlying mechanism that could explain the occurrence of reentrant nematic behavior for polar and non-polar molecules.

## 1. Introduction

The reappearance of a thermodynamic phase as the temperature *T* is lowered (or the pressure *P* is raised) is termed “reentrance”. Reentrant phases are common to many substances, but the first to discover this phenomenon in liquid crystals (LC’s) was Cladis in 1975 [1] for a mixture of two LC molecules with benzene rings and a strongly polar cyano group. Specifically, a nematic phase was found at *T* above the smectic phase (which is the usual) and also below (which was the novelty). This second nematic phase was called *reentrant nematic* (RN).

Cladis already noticed that the spacing between the layers in the smectic phase was not commensurate with the length of the molecule [1], and, because the molecules under investigation were strongly polar, she concluded that association in dimers played a major role in the formation of a RN phase. This idea is consistent with a number of other experiments [2–4]. Indeed, in the compounds studied in those works the polar part is at one molecular end, instead of being in the middle, as for the majority of LC’s. The nonpolar part (aliphatic chain) is at the opposite end of the molecule. This structure will naturally favor a certain degree of dimerization, because the polar groups will preferentially interact with each other through long-range forces, and the nonpolar tails through short-range forces. Cladis *et al.* [3] proposed that it is the short-range interaction between nonpolar tails that stabilizes the smectic layers. It is argued [3,4] that as pressure increases (or equivalently as *T* decreases) the interaction between the polar groups becomes repulsive, and at the same time the nonpolar tails are somewhat compressed, lowering the energy barrier to permeation through the smectic layers. Both effects lead to a destabilization of the smectic order, and hence to the formation of a RN. Later, a RN phase was also found in pure compounds [5–7]. Since then many pure compounds and mixtures have shown to have one or multiple reentrant phases. However, the situation was made more complex by the finding that also terminal-nonpolar substances can exhibit a RN phase [8,9]. Clearly, dimerization cannot be an explanation in this case.

Different theoretical models have been introduced to explain reentrance in LC’s [10]. The “spin-gas theory” was the first microscopic theory of the physical origin of RN phases [11–13]. Those authors consider a system of dipolar molecules interacting via an antiferroelectric interaction. Because the system is fluid, frustration is avoided and dimers can form. However, as *T* is lowered, positional order ensues and the increasing frustration leads to the unbinding of dimers and hence to the RN. Most of the theoretical discussion has focused on polar LC’s forming dimers, because reentrance in LC’s was first found in such systems. However, the fact that also non-polar substances show a RN phase [8,9] hints at the possibility that other mechanisms may lead to a RN as well. Dowell [14] proposed a lattice theory for non-polar single component system that takes into account molecular chain flexibility. In Dowell’s model the smectic phase is formed because of segregated packing of cores and tails. As *T* is lowered, however, the molecular chains become increasingly stiffer, thereby destabilizing the smectic layers. Eventually, it becomes entropically more favorable to disrupt smectic layering such that a RN phase is formed. X-ray [15] and ESR [16] experiments support Dowell’s scenario of reentrance in systems that do not show signs of dimerization [8,9].

A clear agreement whether a single mechanism can explain all reentrant transitions in LC’s is still lacking. Even less clear is the situation of the *dynamical* behavior of reentrant phases. Miyajima *et al.* [17] studied a pure LC substance exhibiting a RN phase with proton NMR experiments. They found no *T*-dependence of the spin-lattice relaxation rate in the nematic phase, but a clear Arrhenius dependence in the RN phase, with an activation energy of 23 kJ mol^−1^, whose dominant contribution is coming from translational diffusion.

Quite opposite results were found by Dong [18–20] for a LC binary mixture. No *T*-dependence was found in the proton NMR spin-lattice relaxation rate in the RN phase, while the nematic phase exhibited an Arrhenius dependence on *T*. Clearly, binary and pure substances have quite different dynamics. With ^129^Xe NMR Bharatan and Bowers [21] studied LC mixtures and found Arrhenius behavior of the spin-lattice relaxation time in both nematic and RN phases. Also, the activation energies appear to be system-dependent. For a binary mixture, the activation energy in the RN is more than two times the value in the N phase, whereas no difference is detected in a ternary mixture [21].

Furthermore, Ratna *et al.* [22] found that the conductivity in the direction parallel to the optical axis is about 16 times larger than the conductivity in the transverse direction for a pure compound in the RN phase, while for a LC mixture the enhancement is about 1.8. Although this is no direct proof of enhanced mass-transport, it hints at the possibility that diffusivity could be enhanced in the RN phase.

From the above discussion it is apparent (to us at least) that, though many different LC systems exhibit reentrant behavior, the molecular mechanism leading to a RN has not uniquely been identified, because the analysis has focused on the molecular details. Some of the present authors recently studied a model system of rod-like LC with molecular dynamics (MD) simulations [23,24]. We showed that as *P* is raised the usual sequence of phase transitions isotropic → nematic → smectic A is followed by a RN phase. This RN is characterized by a stronger orientational order than the conventional nematic phase at lower *P*. However, the fundamental difference lies in the dynamics. The RN phase exhibits a self-diffusion coefficient significantly larger than in the nematic phase. It was shown that the self-diffusion coefficient in the direction of the nematic director varies inversely proportional to the width of the distribution of molecular orientations or, in other words, the decrease in orientational entropy in the RN phase leads to an enhanced diffusivity [23].

A simple structural analysis of molecular configuration provides further insight into the origin of the increased diffusivity in RN’s, by calculating the average molecular distance in the direction transverse to the molecular long axis *r*_⊥_ [24]. A pronounced peak at *r*_⊥_ ≳ 1.0 develops when the system enters the RN phase but is absent otherwise. As *P* increases, this peak grows in magnitude and shifts closer to 1.0. At these intermolecular distances, the molecules are probing the beginning of the repulsive part of their interaction potential. Thus, considering a molecule and its first neighbors, the mutual interactions cancel out and translation motion can be enhanced. Molecules effectively “levitate” in the local, mean molecular potential. A very similar physical situation is encountered in the problem of diffusion in nanoporous materials, such as zeolites. Indeed, a diffusivity maximum is seen in such systems as the size of the diffusing particle reaches a value close to the width of the pore, so that a mutual cancellation of forces occurs [25–27]. This effect is called *levitation* [25–27].

Previously, we have studied the effect of changing *P* on the dynamics of RN’s [23,24]. However, experimentally it is often much easier to change *T* along an isobar than modifying pressure. Also, it is interesting to study the effect of changing the thermal energy of a RN phase to shed some additional light on the dynamical features of RN’s. Here, we perform MD simulations of a model for mesogens, *i.e*., molecules forming LC phases, exhibiting a RN phase for a set of isobars. We find a diffusivity maximum along isobaric cooling. The diffusivity increases dramatically as the system undergoes a smectic A-RN transition and then decreases following an Arrhenius law. These results can help to shed some additional light on the dynamical features of RN’s, because they provide an observable accessible to experimentalists.

Confined LC’s differ from the bulk case both in structure and dynamics. A large number of experimental [28–32] and theoretical works [33–40] have explored many novel aspects of LC under different types of confinement. The reason why confined LC’s behave differently is a combination of (i) the specific interaction with the confining surfaces which effectively introduce an additional external field that couples to the fluid; and (ii) the long-range elastic forces present in ordered LC phases [41]. It is therefore natural to ask whether the enhanced diffusivity in the RN phase is a confinement effect. We have verified that both the presence of the RN phase and the enhanced diffusivity also occur in bulk simulations (not shown here). The only effect of the confining surfaces is a shift of the phase boundaries with respect to the bulk case, that is, the smectic A-RN phase boundary is shifted to lower *P*.

This work is organized as follows. In Section 2 we present our results. In Section 3 we describe our model and computational details of our simulations. Finally, we discuss our results in relation to known experiments in Section 4.

## 2. Results

An isotropic LC has no positional nor orientational order. If its *T* is lowered, orientational symmetry is the first to spontaneously break because of the emergence of a preferential molecular orientation, specified by the nematic director ***n̂***. If *T* is further lowered, positional order breaks next. Layers form in the LC which is now in its smectic phase. Within any single layer molecules have no long-range positional order, which is typical of a liquid whereas in one direction the system exhibits long-range positional order. If the normal to the smectic layers coincides with the nematic director the LC is called smectic A.

We first need to characterize the LC phase by measuring the degree of orientational and translational order. To quantify orientational order we consider the alignment tensor

(1) $$Q\equiv \frac{1}{2N}\sum_{i=1}^{N}\left(3{\hat{u}}_{i}⊗{\hat{u}}_{i}-1\right)$$

where ⊗ indicates the dyadic product and **1** is the unit tensor. See Section 3 for the definitions of the symbols in this Section. Hence, **Q** is a real, symmetric, and traceless second-rank tensor which can be diagonalized. Its largest eigenvalue λ_+_ defines the Maier–Saupe nematic order parameter *S* = 〈λ_+_〉 [42–44] where the angular brackets represent a time average. The eigenvector associated with λ_+_ corresponds to the nematic director ***n̂***. In a macroscopic, bulk isotropic phase *S* = 0 ideally because molecular orientations are randomly distributed while in an ideal nematic phase *S* = 1 because molecules are perfectly aligned with ***n̂***. In any finite size system *S* ≳ 0 for reasons explained elsewhere [45].

Smectic phases are characterized by a density wave breaking the translational symmetry. In their simplest form, smectic A, layers form in the LC fluid with their normal parallel to ***n̂***. A natural definition of an order parameter for a smectic A phase is therefore the leading coefficient of the Fourier series expansion of the density [46]

(2) $$\Lambda \equiv \frac{1}{N}\langle \left|\sum_{i=1}^{N}\text{exp}\left[\frac{2\pi i\;(r_{i}\cdot \hat{n})}{d}\right]\right|\rangle$$

where *d* is the spacing between adjacent smectic layers. In practice, because *d* is not known a priori *d* is adjusted so as to maximize Λ.

**Figure 1:**
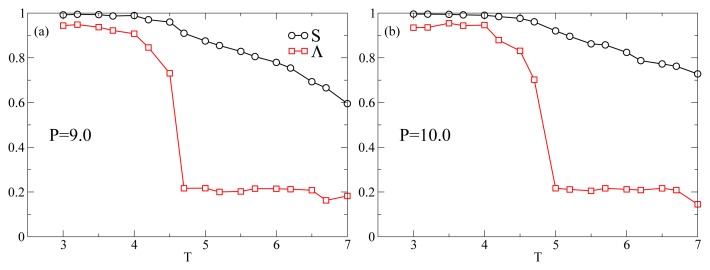

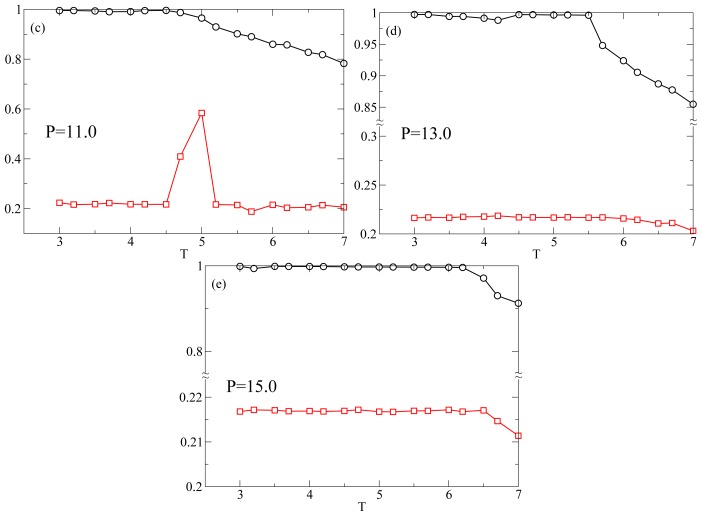
Temperature-dependence of the nematic order parameter *S* (circles) and smectic order parameter Λ (squares) for different isobars. Lines are guides for the eye. All quantities are expressed in standard reduced units (see Section 3).

**Figure 2:**
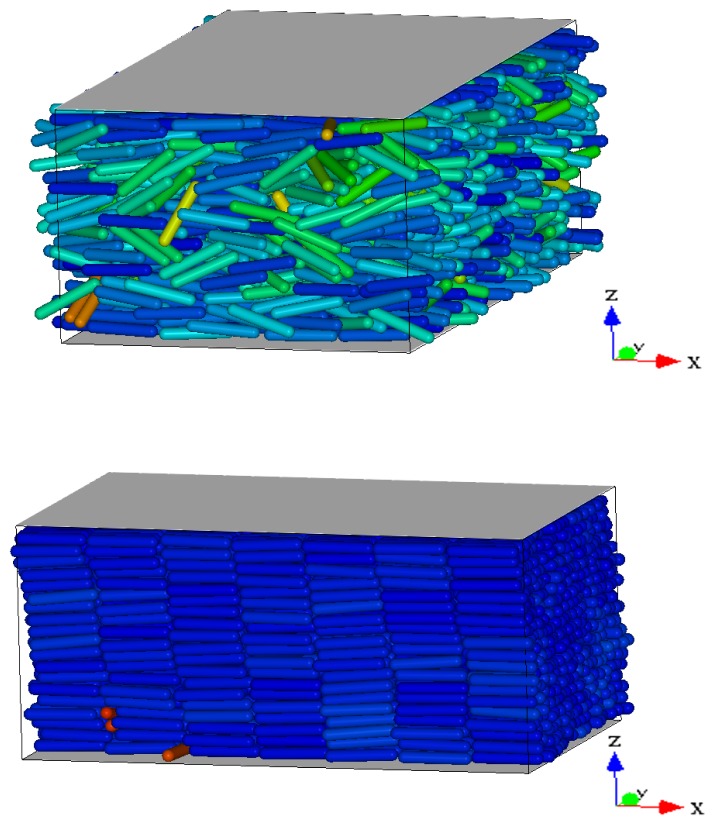

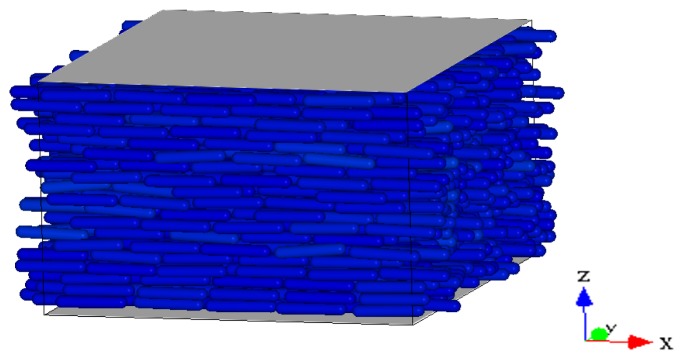
Snapshot of typical configurations of the confined LC system in different phases; grey areas represent the confining walls. From top to bottom we show the nematic (*P* = 11.0, *T* = 6.0), smectic A (*P* = 10.0, *T* = 3.2) and reentrant nematic phase (*P* = 13.0, *T* = 4.0). Graphics generated with the software package qmga [47]. Different colors indicate the degree of alignment to the director, that is blue indicates 0 °, red 90 °, and green intermediate. All quantities are expressed in standard reduced units (see Section 3).

Figure 1 shows the *T*-dependence of the nematic order parameter *S* and the smectic order parameter Λ for different isobars. In this range of *P* at *T* = 7.0 the system is always in the nematic phase as shown by the fact that *S* is always larger than 0.6. The isotropic phase is located at lower values of *P*. At *P* = 9.0 (Figure 1a), *S* increases monotonically reaching values very close to one as *T* decreases. An inspection of Λ reveals that at *T* ≈ 4.5 the system undergoes a discontinuous transition from a nematic phase, characterized by a low value of Λ ≈ 0.2, to a smectic A phase, characterized by Λ ≳ 0.7. At *P* = 10.0 (Figure 1b), *S* and Λ show the same qualitative behavior as in Figure 1a. At *P* = 11.0, while *S* shows the same behavior as for lower *P*, there occurs a qualitative change in the behavior of Λ. It shows a discontinuous increase at *T* = 5.2 corresponding to the nematic–smectic A transition, followed at *T* ≃ 4.5 by a decrease to values characteristic of the nematic phase. Because of the high degree of orientational order and the loss of positional order, the system reenters the nematic phase. The system in the RN phase is therefore characterized by a value of Λ similar to the nematic phase and a value of *S* very close to one. This extremely high value of *S* is due to a combination of finite-size effects and to the simplicity of the interaction potential. Further, at *P* = 13.0 and *P* = 15.0 (Figure 1d,e) Λ never increases above the values characteristic of the nematic phase. The intermittent smectic phase is completely absent at these higher pressures so that the system appears to be always in the nematic phase. However, the *T*-dependence of *S* shows a crossover from a steady increase to a constant at *T_S_*(*P*) ≃ 5.5 for *P* = 13.0 (Figure 1d) and at *T_S_*(*P*) ≃ 6.5 for *P* = 15.0 (Figure 1e). Below we show that even though there is no intermittent smectic phase the dynamical behavior of the system changes strongly at *T_S_*(*P*) so that we can still call RN the phase at *T < T_S_*(*P*). In Figure 2 we show three representative snapshots of the confined LC system in the nematic, smectic A, and RN phase. From these snapshots it is clear that the RN phase has a much larger degree of orientational order than the nematic phase while lacking completely the positional order typical of smectics.

Next, we investigate the dynamics of this system by calculating the mean square displacement (MSD) in the direction of the long molecular axis

(3) $${\langle \Delta r_{\left|\right|}^{2}(\tau )\rangle }_{t}\equiv \frac{1}{N}{\langle \sum_{i=1}^{N}{\left[r_{i}^{\left|\right|}\;(t+\tau )-r_{i}^{\left|\right|}\;(t)\right]}^{2}\rangle }_{t}$$

where *r_i_^||^* ≡ ***û****_i_* · ***r****_i_* and the subscript *t* indicates an average over initial time origins, which is a consequence of the stationary character of temporal correlations in equilibrium systems [48]. Because we are considering phases for which there is already a preferential global orientation, this definition of MSD captures the motion along the nematic director.

**Figure 3:**
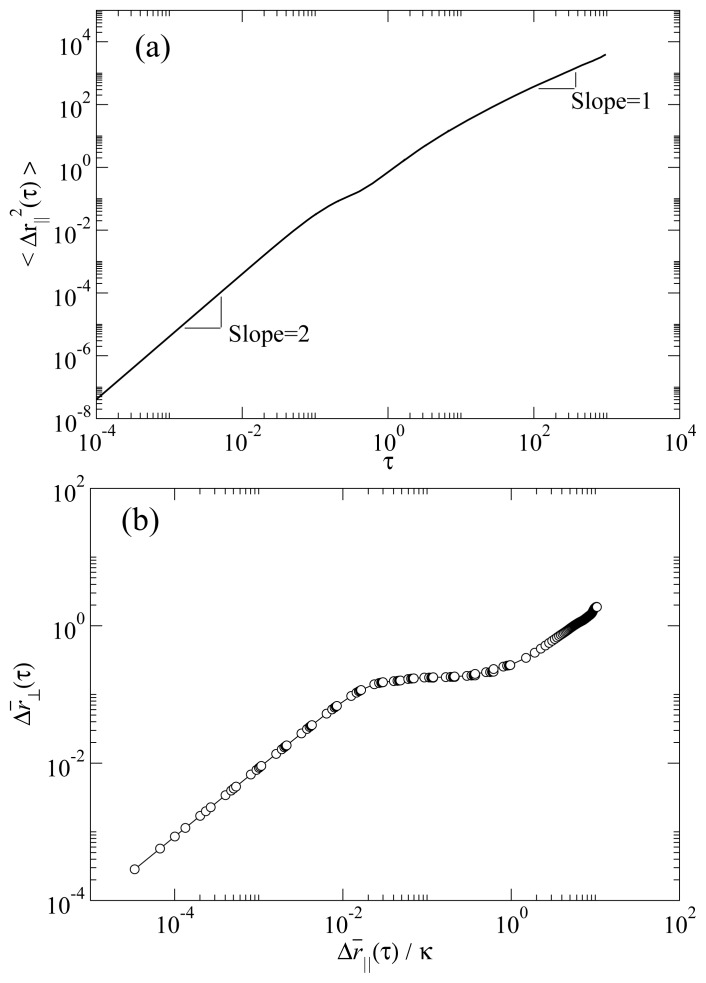
(**a**) Typical mean square displacement in the reentrant nematic phase. The curve shows the two characteristic regimes: ballistic regime at short time scales, where the curve has a slope of two, and the diffusive regime at long time scales, where the curve has a slope of one; (**b**) Root mean square displacement in the direction perpendicular to the molecular long axis *vs*. the root mean square displacement in the direction parallel to the molecular axis in units of the aspect ratio *κ*. Data in both panels are from simulations at *P* = 13.0 and *T* = 4.0. All quantities are expressed in standard reduced units (see Section 3).

Figure 3a shows the parallel MSD in a typical RN phase. Two characteristic temporal regimes can be clearly recognized. At short times the curve has a quadratic temporal dependence, a clear signature of the ballistic regime. At long times the MSD has a linear time dependence, characteristic of the diffusive regime. We note that the temporal dependence of the MSD excludes the possibility that this phase were a columnar rather than RN. This is because a columnar phase would necessarily exhibit single-file diffusion, which scales with time as *t*^1/2^. The MSD in the direction perpendicular to the molecular axis 〈Δ*r*_⊥_^2^ (*τ*)〉*_t_* is obtained by replacing *r_i_^||^* with ***r****_i_*^⊥^ ≡ ***r****_i_* − (***û****_i_* · ***r****_i_*) ***û****_i_* in Equation 3. In Figure 3b we show the root MSD for perpendicular versus parallel molecular displacements $\Delta {\bar{r}}_{⊥,||}\equiv \sqrt{{\langle \Delta r_{⊥,||}^{2}\;(\tau )\rangle }_{t}}$. It is apparent that both parallel and perpendicular MSD reach the diffusive regime, that is, when a molecule on average has moved many times its length in the parallel direction, it will have moved also a number of times its diameter in the perpendicular direction [24]. Hence, we do not observe any dynamical behavior consistent with the existence of a columnar phase.

From the long time behavior of the parallel MSD we extract the self-diffusion coefficient through Einstein’s relation

(4) $$D_{\left|\right|}=\underset{\tau \to \infty }{\text{lim}}\frac{1}{2\tau }{\langle \Delta r_{\left|\right|}^{2}\;(\tau )\rangle }_{t}$$

**Figure 4:**
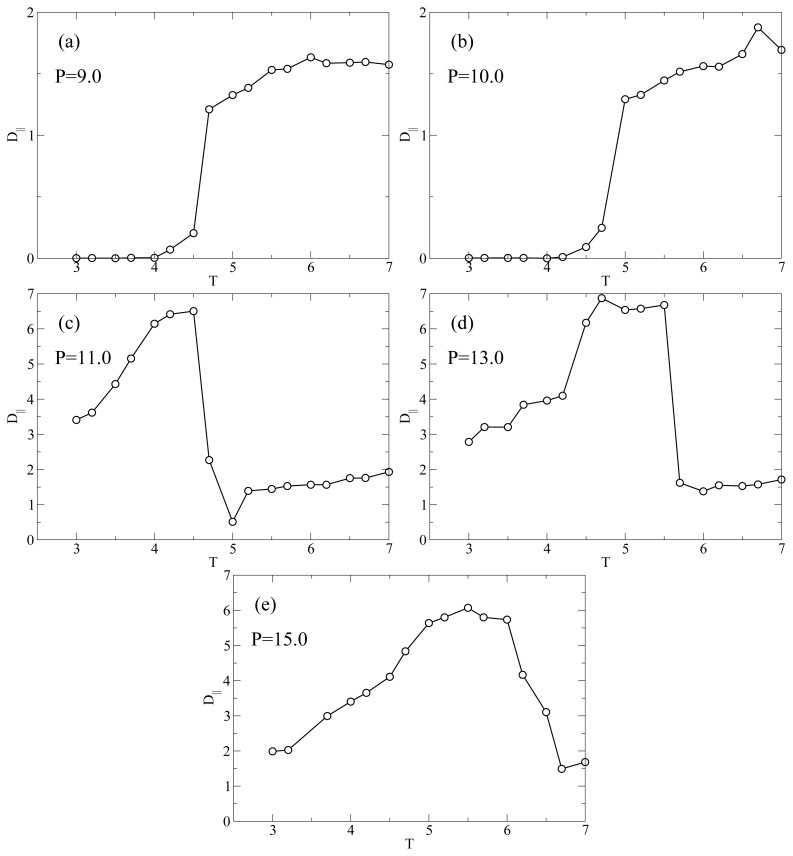
Temperature-dependence of the parallel diffusivity *D_||_* for different isobars. Lines are guides for the eye. All quantities are expressed in standard reduced units (see Section 3).

Figure 4 shows the *T* behavior of *D_||_* for the different isobars studied here. At *P* = 9.0 (Figure 4a) the diffusivity has a value of *D_||_* ≈ 1.5 at high *T* in the nematic phase; then, it exhibits a discontinuous drop to very low values *D_||_* ≈ 10^−3^ upon entering the smectic phase. From the parallel plot in Figure 1a it is evident that this drastic drop occurs upon entering the smectic phase at *T* ≈ 4.5. It is clearly due to the hindrance to translational diffusion caused by the smectic layers. Figure 4b shows that the isobar at *P* = 10.0 has the same qualitative behavior as the isobar at *P* = 9.0, that is a value *D_||_* ≈ 1.5 in the nematic phase followed by a drop at *T* = 5.0 (see Figure 1b) to very small values in the smectic phase. At *P* = 11.0 (Figure 4c) *D_||_* has a different *T*-dependence. In the nematic phase (large *T*) *D_||_* ≃ 1.5 as at lower *P*; at *T* = 5.0 there is a drop in *D_||_* due to the formation of smectic layers. We note that *D_||_* ≃ 0.5 indicating that the smectic phase at *P* = 11.0 has a higher mobility than the one at lower *P*. This observation is in agreement with the lower value of Λ at *P* = 11.0 (see Figure 1c) for the smectic phase with respect to its value at *P* = 9.0 or *P* = 10.0.

However, at *T* = 4.7 there is a sudden jump in *D_||_* when the RN phase forms. We define this transition temperature *T_D_*(*P*). *D_||_* reaches a maximum value approximately equal to 6.5 which is considerably larger than the typical diffusivity in the nematic phase. As *T* is further lowered*D_||_* decreases monotonically. Below we analyze the *T*-dependence of *D_||_* in the RN phase. The dramatic increase in self-diffusion in the direction of ***n̂*** in combination with nearly perfect nematic order prompted us to refer to liquid crystals in the RN phase as “supernematics” [23]. At *P* = 13.0 (Figure 4d) *D_||_* ≈ 1.5 in the interval 6.0 ≤ *T* ≤ 7 which corresponds to a typical value in the nematic phase. At *T* = *T_D_*(*P*) ≃ 5.5 the diffusivity jumps to almost a value of 7.0, which is a typical value in the RN phase. Figure 4d is interesting because it shows a sharp and distinct jump in *D_||_* even though there is no intermittent smectic phase such that the system is always in a nematic state. If we compare with Figure 1d, we realize that *S* shows a small jump at *T* = *T_S_* ≃ 5.5. The coincidence of *T_S_*(*P*) and *T_D_*(*P*) indicates that even though these two states are dynamically distinct, structural differences between nematic (at high *T*) and RN (at low *T*) are very subtle. Finally, Figure 4e indicates that at *P* = 15.0 *D_||_* has a qualitatively similar behavior compared with the case of *P* = 13.0. Also for *P* = 15.0, the small jump in *S* occurs at the *T* = *T_S_* (Figure 1e) which coincides with *T* = *T_D_* where *D_||_* has a large increase (Figure 4e).

**Figure 5:**
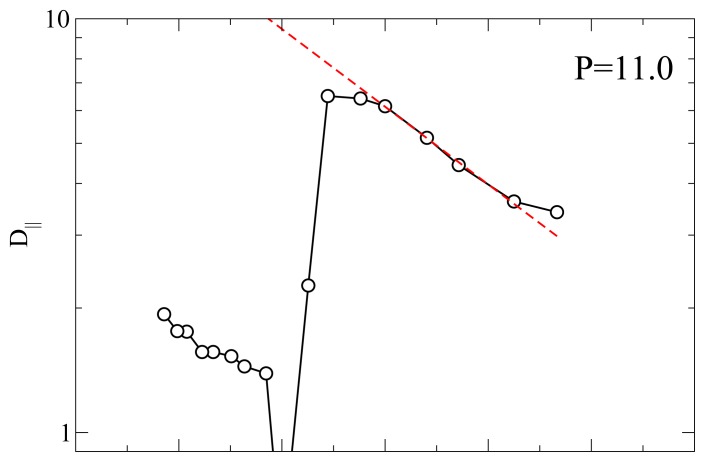

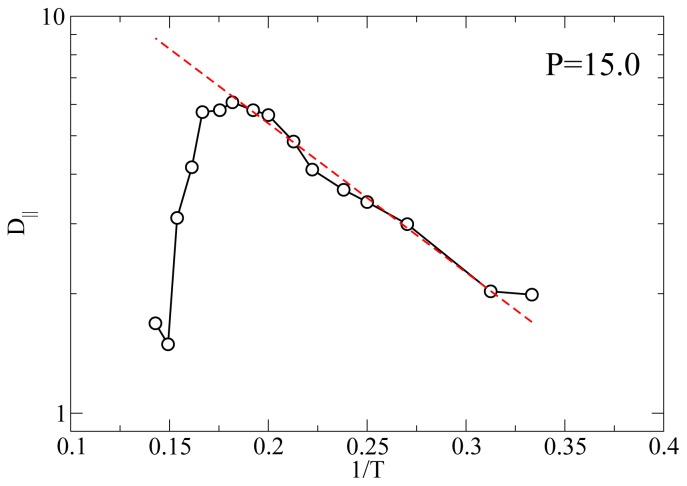
Arrhenius plot of the parallel diffusivity *D_||_* at *P* = 11.0 (top panel) and *P* = 15.0 (bottom panel). The dashed line is a fit of the low *T* values to an Arrhenius equations. Both fits yield an activation energy *E_A_* = 8.64 (corresponding to about 0.3 eV). Solid lines are guides for the eye. All quantities are expressed in standard reduced units (see Section 3).

Another useful characterization of the RN phase is possible through its activation energy for the diffusion process because it can be measured with many different experimental techniques such as QENS and NMR. Figure 5 shows an Arrhenius plot of *D_||_* at *P* = 11.0 and *P* = 15.0. At *P* = 11.0 and for *T* below the smectic A-RN transition the calculated *D_||_* appears to follow an Arrhenius dependence, that is

(5) $$D_{\left|\right|}=D_{0}\;\text{exp}(-E_{A}/k_{\text{B}}T).$$

From a best-fit of the MD results for *D_||_* we obtain an activation energy *E_A_* ≃ 8.64 at both *P* = 11.0 and *P* = 15.0. This value of *E_A_* corresponds to 29.2 kJ mol^−1^ if we choose *ε* = 0.56127 *×* 10^−20^ J [49] (see also Ref [50]). As discussed elsewhere [23] there are only a few experimental investigations of the dynamics of RN’s. For example, for the activation energy of the dielectric relaxation frequency Ratna *et al.* [51] found a value of 0.457 eV or higher, depending on the mixture; our value *E_A_* = 0.3 eV is slightly lower. In proton NMR experiments on a pure LC exhibiting a RN phase Miyajima *et al.* [17] measured an activation energy of 23 kJ mol^−1^ which turns out to be a bit lower than our result. The fact that our calculation of *E_A_* falls in between these experimental measurements is gratifying given the simplicity of the geometrical shape and of the interaction between the mesogens.

**Figure 6:**
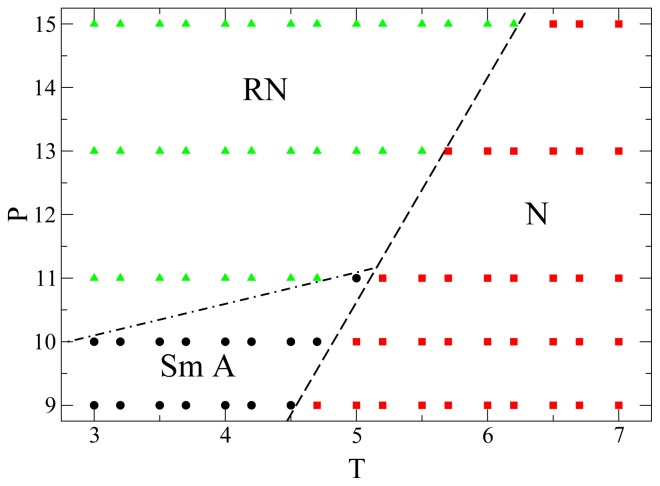
Phase diagram of the GBK model of mesogens. State points belonging to different phases are indicated as follows: Smectic A with circles, nematic with squares and RN with triangles. Dashed lines are guides for the eye indicating the approximate position of phase boundaries. All quantities are expressed in standard reduced units (see Section 3).

Finally, in Figure 6 we show the portion of *P* − *T* phase diagram here obtained for the mesogens. We identify the phase of any individual state point through structural order parameters and also through the dynamic behavior. Although for *P* > 11.0 there is no intermittent smectic phase separating the nematic and the RN phases, and there is only a very subtle structural difference between nematic and RN, these two phases nevertheless exhibit a remarkably different dynamic behavior. The lines separating the different phases are only rough indications of the phase boundaries. To determine accurately these phase boundaries, by calculating the free energies for example, is beyond the scope of the present work. Over the range of *P* and *T* considered in Figure 6 no isotropic phase occurs, because it is located at lower *P* or higher *T*. At low *P* only nematic and smectic phases are present, whereas at higher *P* the smectic phase gives way to the RN phase. For *P >* 11.0 there is only a transition between nematic and RN. The three phases meet at a triple-point approximately located at *P* ≃ 11.0 and *T* ≃ 5.0. Preliminary simulations show that the qualitative features of this phase diagram do not depend on system size. However, the phase boundaries do shift as *N* increases which is a well-known finite-size effect [52,53].

## 3. Model

A popular model to simulate mesogens is the Gay–Berne potential [54]. A drawback of this potential is that Gay–Berne molecules have an ellipsoidal core, which is somewhat unrealistic in terms of the chemical structure of liquid-crystalline organic compounds. A more appropriate shape that still retains computational simplicity is a spherocylinder [55,56], which is defined as a cylinder of length *L* and diameter *σ* capped at both ends with hemispheres of diameter *σ* (the total length of the spherocylinder is therefore *L* + *σ*). Here, we consider the Gay–Berne–Kihara (GBK) model for prolate mesogens [56]. The GBK model potential conveniently preserves the anisotropic interaction of the Gay–Berne fluid and includes also a spherocylindrical molecular shape.

The fluid–fluid interaction between a pair of GBK molecules *i* and *j* depends on the molecular orientations represented by unit vectors ***û****_i_* and ***û****_j_*, respectively, and their distance ***r****_ij_* ≡ ***r****_i_* − ***r****_j_*, that is

(6) $$u_{\text{ff}}=4{ɛ}_{\text{ff}}({\hat{r}}_{ij},{\hat{u}}_{i},{\hat{u}}_{j})\left[{\left(\frac{\sigma }{d_{ij}^{\text{m}}}\right)}^{12}-{\left(\frac{\sigma }{d_{ij}^{\text{m}}}\right)}^{6}\right]$$

where ***r̂****_ij_* ≡ ***r****_ij_/r_ij_*, *r_ij_* ≡ |***r****_ij_*|, and the function *d_ij_*^m^(***r****_ij_,* ***û****_i_,* ***û****_j_*) is the *minimum* distance between the central axes of two mesogens [57]. The orientation-dependent interaction strength in Equation 6 may be cast as

(7) $${ɛ}_{\text{ff}}({\hat{r}}_{ij},{\hat{u}}_{i},{\hat{u}}_{j})=ɛ{\left\{1-\frac{\chi^{\prime }}{2}\left[\frac{{\left({\hat{r}}_{ij}\cdot {\hat{u}}_{i}+{\hat{r}}_{ij}\cdot {\hat{u}}_{j}\right)}^{2}}{1+\chi^{\prime }{\hat{u}}_{i}\cdot {\hat{u}}_{j}}+\frac{{\left({\hat{r}}_{ij}\cdot {\hat{u}}_{i}-{\hat{r}}_{ij}\cdot {\hat{u}}_{j}\right)}^{2}}{1-\chi^{\prime }{\hat{u}}_{i}\cdot {\hat{u}}_{j}}\right]\right\}}^{2}\times \frac{1}{\sqrt{1-\chi^{2}{\left({\hat{u}}_{i}\cdot {\hat{u}}_{j}\right)}^{2}}}$$

where the parameters *χ* and *χ*′ are given by

(8a) $$\chi \equiv \frac{\kappa^{2}-1}{\kappa^{2}+1}$$

(8b) $$\chi^{\prime }\equiv \frac{\sqrt{\kappa^{\prime }}-1}{\sqrt{\kappa^{\prime }}+1}$$

In these last two expressions parameters *κ* = *L* + *σ*, and *κ*′ = 5 represents the interaction strength for a side-side relative to an end-end configuration of a pair of spherocylinders.

We confine the LC system along the *z* direction with two planar walls arranged in a slit-pore geometry. We model the fluid-substrate interaction between mesogen *i* and wall *k* via a Lennard-Jones potential, effectively integrated over a flat surface (up to numerical factors), because we consider atomically smooth confining surfaces such that

(9) $$u_{\text{fs}}=4{ɛ}_{\text{fs}}\rho_{\text{s}}\left[{\left(\frac{\sigma }{d_{ik}^{\text{m}}}\right)}^{10}-{\left(\frac{\sigma }{d_{ik}^{\text{m}}}\right)}^{4}g({\hat{u}}_{i})\right]$$

the index *k* = 1, 2 refers to the substrate. We choose a strength of interaction *ε*_fs_ = *ε* and $\rho_{\text{s}}=2\pi /\sqrt[{3}]{2}$ is the areal density of a single layer of atoms arranged according to the (100) plane of a face-centered cubic lattice, and *d_ik_*^m^ is the minimum distance between molecule *i* and wall *k*. The diameter *σ* of these substrate atoms is taken to be the same as the diameter of a spherocylinder of the confined fluid phase. The function *g*(***û****_i_*) in Equation 9 is the “anchoring function”, which introduces a dependence of the fluid-substrate interaction on the molecular orientation relative to the wall. The functional form of Equation 9 allows to easily select a preferential anchoring, while maintaining computational simplicity (other choices are possible, of course; see, e.g., [38,58,59]). Specifically, we choose degenerate planar anchoring [60]

(10) $$g(\hat{u})={\left(\hat{u}\cdot {\hat{e}}_{\text{x}}\right)}^{2}+{\left(\hat{u}\cdot {\hat{e}}_{\text{y}}\right)}^{2}$$

which favors orientations lying in the plane of the wall where ***ê***_x_ and ***ê***_y_ are the unit vectors of the *x* and *y* axis, respectively.

All quantities are expressed in the standard reduced units, that is, we use *σ* as the unit of length, *ε* as the unit of energy, and the mass of a spherocylinder *m* as the unit of mass. From these choices follows that time is expressed in units of (*mσ*^2^*/ε*)^1/2^, *T* in units of *ε/k*_B_ where *k*_B_ is the Boltzmann constant, *P* in units of *ε/σ*^3^, and diffusivity in units of (*εσ*^2^*/m*)^1/2^.

We perform MD simulations of *N* = 3000 GBK molecules with *L* = 5 in the microcanonical ensemble because we do not want to perturb the true microscopic dynamics of the fluid. We employ the velocity-Verlet algorithm for elongated molecules [49]. At high pressures, when the dynamics is sluggish, long simulation runs (up to 2 *×* 10^7^ steps) are necessary to access the diffusive regime. However, microcanonical MD simulations are plagued by drifts in the total energy (which should strictly be conserved on account of the underlying physical principles) because of the accumulation of numerical errors. To limit this problem, we choose a rather small integration step Δ*t* = 10^−4^. Further, to speed up our simulations we parallelize the computation of molecular forces in our algorithm with OpenMP directives.

Preliminary runs to equilibrate the system at the desired *T* and *P* were performed in the NPT ensemble by using a Nosé–Hoover thermostat and a Hoover barostat [49] that allows for independent variations of the simulation box side-length in the *x* and *y* direction to accommodate anisotropic phases and to avoid spurious mechanical stresses [61]. Once a number of observables such as average energy, volume, and the components of the pressure tensor reach a stationary regime we perform microcanonical simulations using the equilibrated configurations as initial configurations. Finally, in all our simulations the confining walls are separated by a fixed distance *s_z_* = 19.

## 4. Conclusions

We present MD simulations of a LC system confined by two parallel atomically smooth walls. We employ the GBK model for rod-like mesogens [56]. This model has the same anisotropic interaction strength as the well-known Gay–Berne model [54] but differs from the latter because the shape of the mesogen is a spherocylinder and not an ellipsoid of revolution as in the Gay–Berne model. The GBK model exhibits a RN phase in addition to the most common LC phases, such as isotropic, nematic and smectic A. The RN phase is characterized by large orientational order and by enhanced diffusivity. In past works [23,24] we have studied how the nematic and smectic order parameters and the diffusivity depend on *P*. Here, we studied the *T*-dependence of the order parameters and also characterize the dynamics of the system through the self-diffusion coefficient. We observe a diffusivity maximum by varying *T* along isobars upon entering the RN phase. The diffusivity increases rapidly by an order of magnitude when the RN phase forms and then decreases following an Arrhenius law. We determine an activation energy *E_A_* = 29.2 kJ mol^−1^ for the diffusion process which falls within the range of the few values reported experimentally that are available for the dynamics of RN’s in pure compounds and binary mixtures. Furthermore, considering the absence of single-file diffusion in the dynamics of the system, we can exclude the possibility that the phase with enhanced diffusivity is a columnar phase rather than reentrant nematic.

Our calculations apply to the class of non-polar LC molecules exhibiting RN behavior because the GBK model does not include molecular dipole moments. A natural question to ask could be: Is there any similarity between the mechanism leading to reentrance in polar LC’s and what we have found for non-polar LC’s? We think the answer might be yes. Cladis and collaborators point out that repulsive forces are responsible for the disruption of smectic layers in polar LC exhibiting a RN phase [3,4]. These polar LC molecules tend to form dimers because of their antiferroelectric interactions. However, as *P* increases or *T* decreases and their arrangement is increasingly compact, the interaction eventually turns repulsive and destabilizes smectic layers: Hence, a RN forms. Repulsive interactions between flexible tails in a LC molecule play a dominant role in destabilizing the smectic layers and therefore in producing a RN phase also in a model of non-polar molecules [14,62]. This is supported by our finding that the average distance between neighboring molecules in the direction perpendicular to their long axis is very close to one molecular diameter [24]. From this it follows that

- the local potential energy landscape must be rather flat with only shallow minima, thus disrupting the attraction that stabilizes smectic layers, and
- the mutual cancellation of forces between neighboring molecules leads to an effect analogous to levitation in porous media, which can explain the enhanced diffusivity characterizing RN’s. Hence, we conclude that repulsive interactions may explain reentrance in the different physical situations mentioned above.

Both the existence and the features of the RN phase are not affected by the presence of confining walls. We have verified that the only effect of the confining surfaces is a shift of the phase boundaries with respect to the bulk case, that is the smectic A-RN phase boundary is shifted to lower *P*.
